# Tinea Corporis and Tinea Pedis Can Masquerade As Other Cutaneous Conditions in Decedents: Forensic Dermatology of Dermatophyte Infections

**DOI:** 10.7759/cureus.101452

**Published:** 2026-01-13

**Authors:** Philip R Cohen, Katie F Hanson, Joseph A Prahlow

**Affiliations:** 1 Dermatology, Davis Medical Center, University of California, Sacramento, USA; 2 Dermatology, College of Osteopathic Medicine, Touro University California, Vallejo, USA; 3 Maples Center for Forensic Medicine, College of Medicine, University of Florida, Gainesville, USA; 4 Pathology, St. Louis University School of Medicine, St. Louis, USA; 5 Forensic Medicine, Office of the Medical Examiner, St. Louis, USA

**Keywords:** acquired, corporis, cutaneous, dermatology, dermatophyte, forensic, ichthyosis, pedis, skin, tinea

## Abstract

Decedents can have lesions of their skin, mucosa, hair, or nails that may be related to their cause of death; alternatively, these lesions may be coincidentally present and not have associated forensic implications. Superficial fungal infection of the skin, such as tinea corporis and tinea pedis, can mimic other cutaneous conditions, including diseases, neoplasms, or infection. Two decedents had superficial fungal infections that mimicked other conditions, and the cutaneous presentation of their dermatophyte infection was selected to be reported based on their illustrative value. Dermatophyte infections of the body can present with extensive involvement of the skin; a decedent had diffuse tinea corporis that clinically mimicked psoriasis vulgaris, dermatitis, and cutaneous T-cell lymphoma; the diagnosis of a fungal infection of the skin was established after microscopic examination of lesional skin biopsies. The fungal organisms could not be readily visualized on the hematoxylin and eosin-stained sections; however, the fungal hyphae were easily observed after the sections were stained with periodic acid-Schiff stain. Another decedent had two different acquired skin conditions; the first appeared as cutaneous plaques; the differential diagnosis included ichthyosis, dermatitis, and tinea corporis. Examination of the epidermis from a skin biopsy showed the absence of both the granular layer and fungal hyphae on hematoxylin and eosin-stained sections, and the periodic acid-Schiff stain did not demonstrate any fungal organisms; this confirmed the suspected diagnosis of acquired ichthyosis. The second skin disease appeared as severe hyperkeratosis of the soles; the plantar lesions prompted the consideration of secondary syphilis plantar lesions; serologic evaluation was negative for spirochetal infection, and the periodic acid-Schiff-stained sections of the plantar skin biopsy showed fungal hyphae, establishing the diagnosis of hyperkeratotic (moccasin-type) tinea pedis. In summary, a cutaneous dermatophyte infection can morphologically present like a skin condition, a cutaneous malignancy, or an infection with mucocutaneous symptoms. In addition, more than one skin condition may be concurrently present in a decedent. Therefore, in conclusion, the forensic pathologist should entertained the possibility of performing a skin biopsy of any papulosquamous skin lesion on a decedent; importantly, the microscopic evaluation of the tissue section should not only be stained with hematoxylin and eosin but also with a stain which readily allows superficial fungal hyphae to be observed in the stratum corneum of the epidermis such as periodic acid-Schiff stain.

## Introduction

Dermatophytes are superficial fungal skin infections from the following species: Trichophyton, Microsporum, and Epidermophyton. They can affect the skin, hair, and/or nails; clinical manifestations include papulosquamous cutaneous lesions, inflammatory or non-inflammatory alopecia, and onychomycosis with nail plate dyschromia and dystrophy. Their derivation can be anthrophilic, geophilic, or zoophilic [1-6].

Ichthyosis vulgaris can be inherited (an autosomal semidominant condition with incomplete penetrance) or acquired. Morphologically, it presents as papulosquamous plaques. Acquired ichthyosis can be a malignancy-related cutaneous paraneoplastic syndrome associated with or potentially associated with numerous systemic conditions, such as sarcoidosis, or a drug-related adverse cutaneous side effect [7-13].

Two decedents had dermatophyte infections that mimicked other conditions [14-16]. The cutaneous presentation of their superficial fungal skin infections was selected because of their illustrative value to be reported. One of the men had extensive and diffuse tinea corporis that appeared like other papulosquamous skin conditions or cutaneous lymphoma. The other man had both leg lesions of acquired ichthyosis and plantar lesions of tinea pedis; the latter were clinically suggestive of conditions such as dermatitis or secondary syphilis.

Superficial dermatophyte skin infections can mimic other cutaneous conditions. The clinical differential diagnoses of tinea corporis and tinea pedis are discussed; the diseases that can be associated with acquired ichthyosis are also summarized. The clinical and forensic significance of differentiating cutaneous fungal infections from other skin conditions, tumors, or infections has both clinical and forensic importance; dermatologic findings at autopsy may be the cause of death of the decedent or can be a contributing etiologic factor to the victim dying [17,18]. Therefore, a skin biopsy should be considered for the evaluation of a papulosquamous skin condition in a decedent.

## Case presentation

Case 1

A 59-year-old Black man was found dead on the front porch of a home in the early evening. The ambient temperature was 18.7 °F; the temperature that day ranged from 4 to 30 °F. The ambient temperature on the two prior days had ranged from 0 to 18 °F.

The man had been wearing several layers of clothing. When he was discovered, his face and extremities appeared to be frozen; however, his abdomen was still warm and pliable. Cutaneous examination of exposed skin revealed a frozen, whitish-colored substance about the nares. The internal temperature of the decedent was 60.5°F; it was obtained using a digital thermometer that had been inserted into a small incision in the upper right quadrant of the abdomen and into the liver.

A complete autopsy was performed. Postmortem changes were present. Livor mortis was present as fixed, posteriorly distributed purple-red lividity. Rigor mortis was moderate to severe and symmetric.

**Figure 1:**
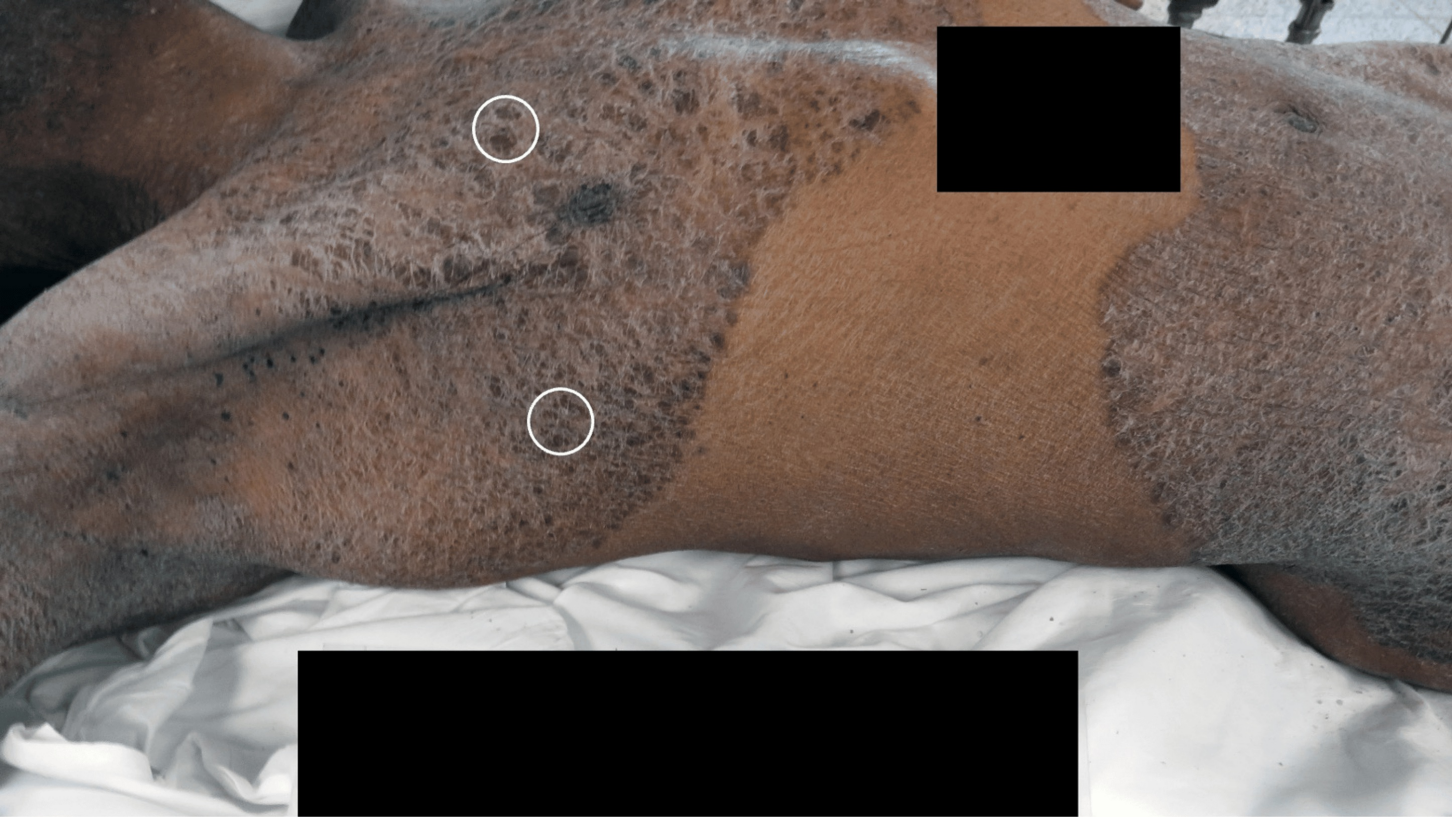
Tinea corporis presenting as extensively distributed hyperpigmented plaques in Case 1 A 59-year-old Black decedent whose immediate cause of death was hypertensive and atherosclerotic cardiovascular disease exacerbated by acute cocaine use and whose manner of death was accidental. Hyperpigmented plaques are symmetrically distributed on the anterior and lateral chest, extending onto the neck and axillae; some of the darker plaques are outlined by white ovals. The confluent plaques also extend from the lower abdomen onto the legs. Black rectangles obscure identifying information. Psoriasis, dermatitis, and cutaneous lymphoma are included in the clinical differential.

A comprehensive skin examination was conducted. Symmetric hyperpigmented plaques were present diffusely on his body. Plaques were present on the anterior and lateral chest extending onto the neck and axillae; a small area of normal-appearing abdominal skin was noted, and similar confluent plaques extended from above the umbilicus onto the legs (Figure 1).

**Figure 2:**
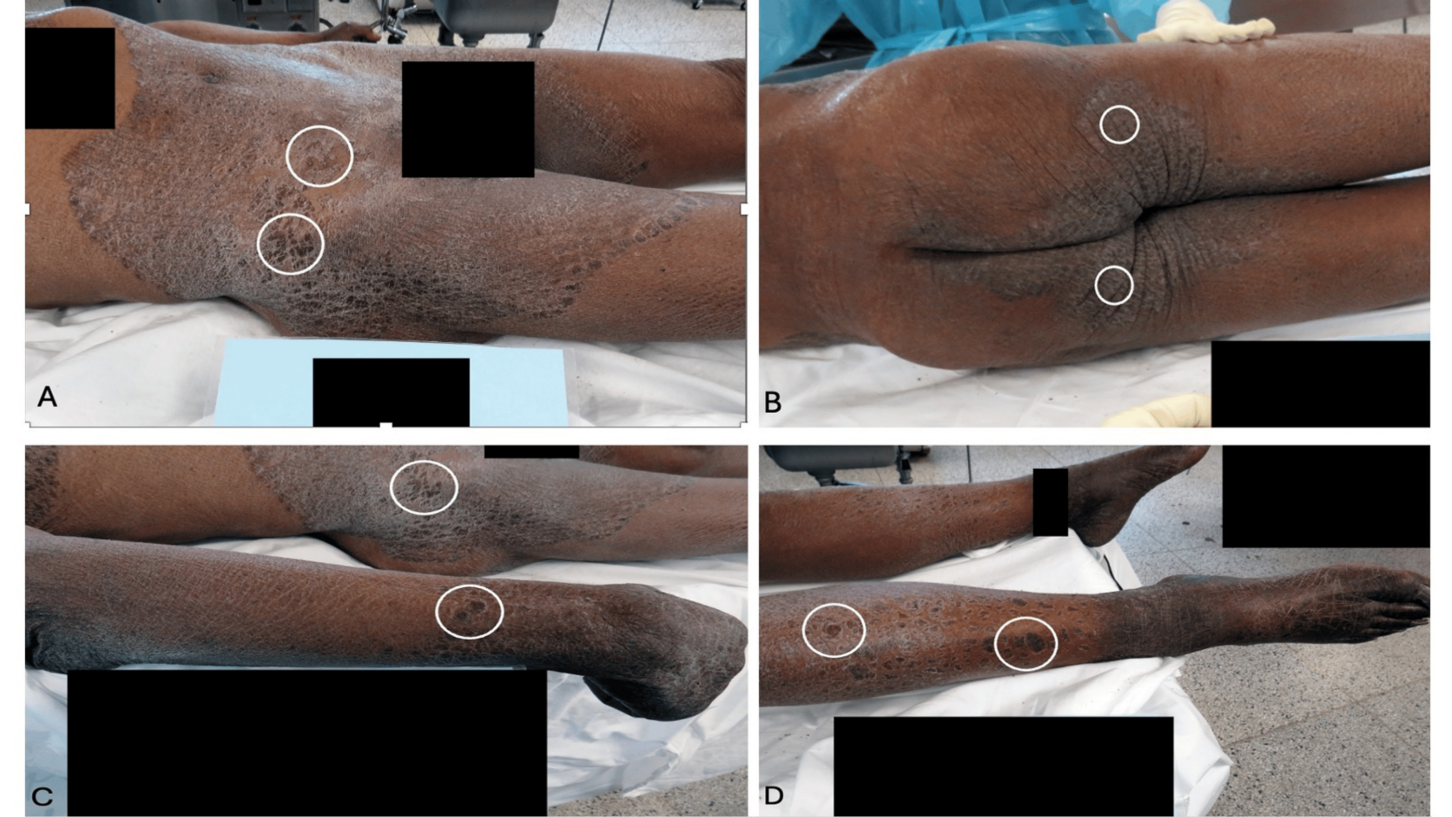
Additional views of extensive tinea corporis in Case 1 Confluent hyperpigmented plaques from the lower abdomen extend to the lateral, anterior, and medial thighs (A). Darker plaques are surrounded by white ovals (A-D). Black rectangles cover the genitalia (A) and obscure identifying information (A-D). The keratotic plaques involve the intergluteal cleft, the medial buttocks, and the posterior and medial areas of the proximal thighs (B). The lateral thigh shows hyperkeratotic plaques (C); the distal extensor forearm (C) and the pretibial legs (D) also demonstrate similar lesions. The pigmented plaques are demonstrated on the dorsal hands (C) and feet (D). The extensive tinea corporis was a coincidental finding that did not contribute to the cause of death of this individual.

The plaques were present on the entire proximal thighs, the intergluteal cleft, the medial buttocks, the distal extensor forearm, the pretibial legs, and the dorsal hands and feet (Figure 2); the toenails were long and dystrophic. In summary, the man had dark plaques that were extensively distributed on his body. Initially, the clinical differential included psoriasis, dermatitis, and cutaneous lymphoma; tinea corporis was added to the list of possible diagnoses after a forensic dermatology consultation.

**Figure 3:**
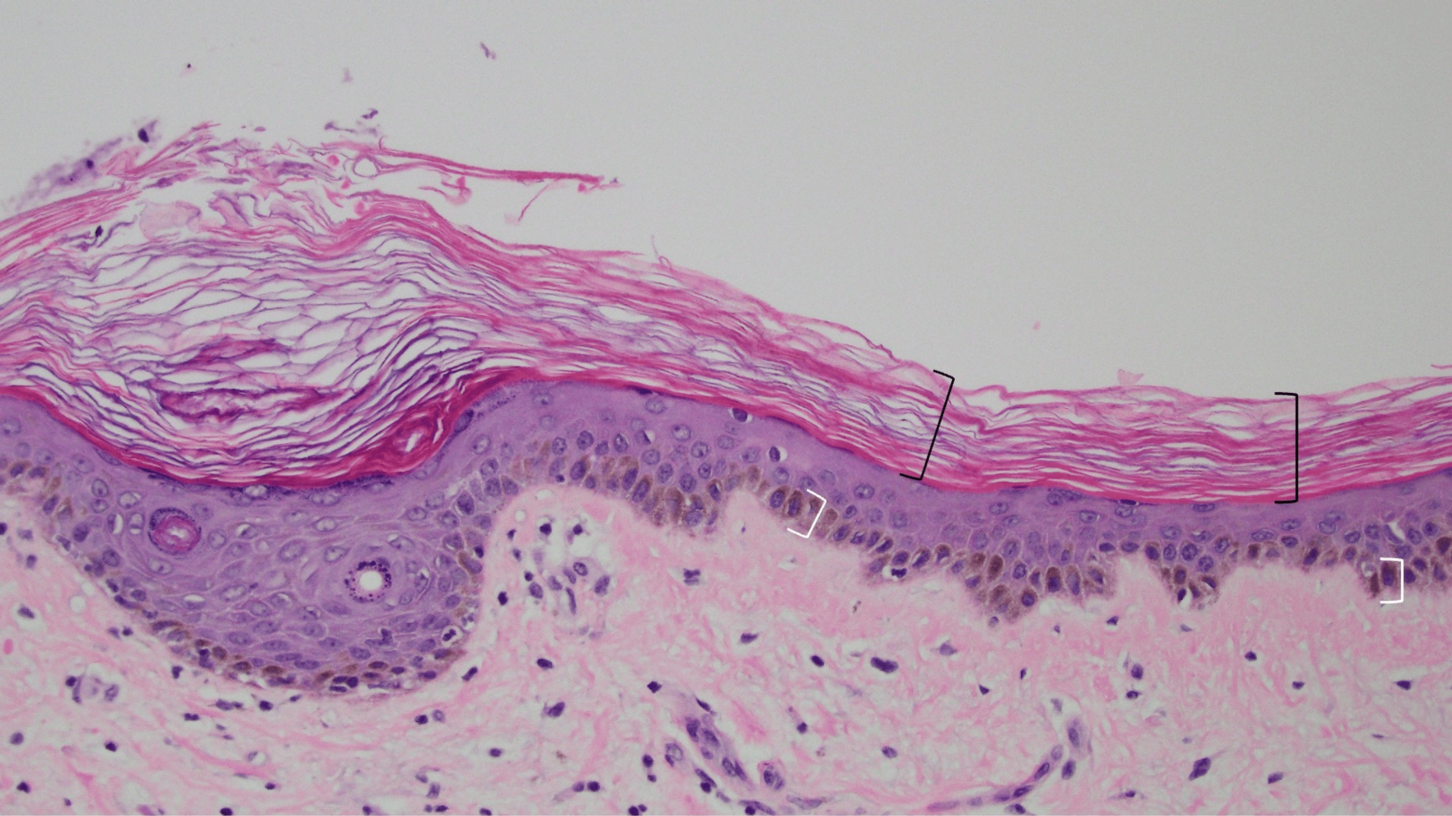
Hematoxylin and eosin-stained slides of extensive tinea corporis in Case 1. There is a basket-weave-like thickening of the stratum corneum, and keratinocyte nuclei are not retained; the changes of orthokeratosis are demonstrated within the black open bracket. Consistent with the decedent’s race, there is hyperpigmentation of the basal layer, shown within the white open bracket. There is no inflammatory infiltrate in the dermis. Fungal hyphae cannot be readily observed (hematoxylin and eosin stain, x20).

A plaque on the chest was biopsied. Microscopic changes on the hematoxylin and eosin-stained sections showed a basket-weave-like thickening of the stratum corneum without retention of keratinocyte cell nuclei (orthokeratosis). There was hyperpigmentation of the basal layer (consistent with the decedent’s race), the thickness of the epidermis was not increased, and there was no inflammation in the dermis (Figure 3).

**Figure 4:**
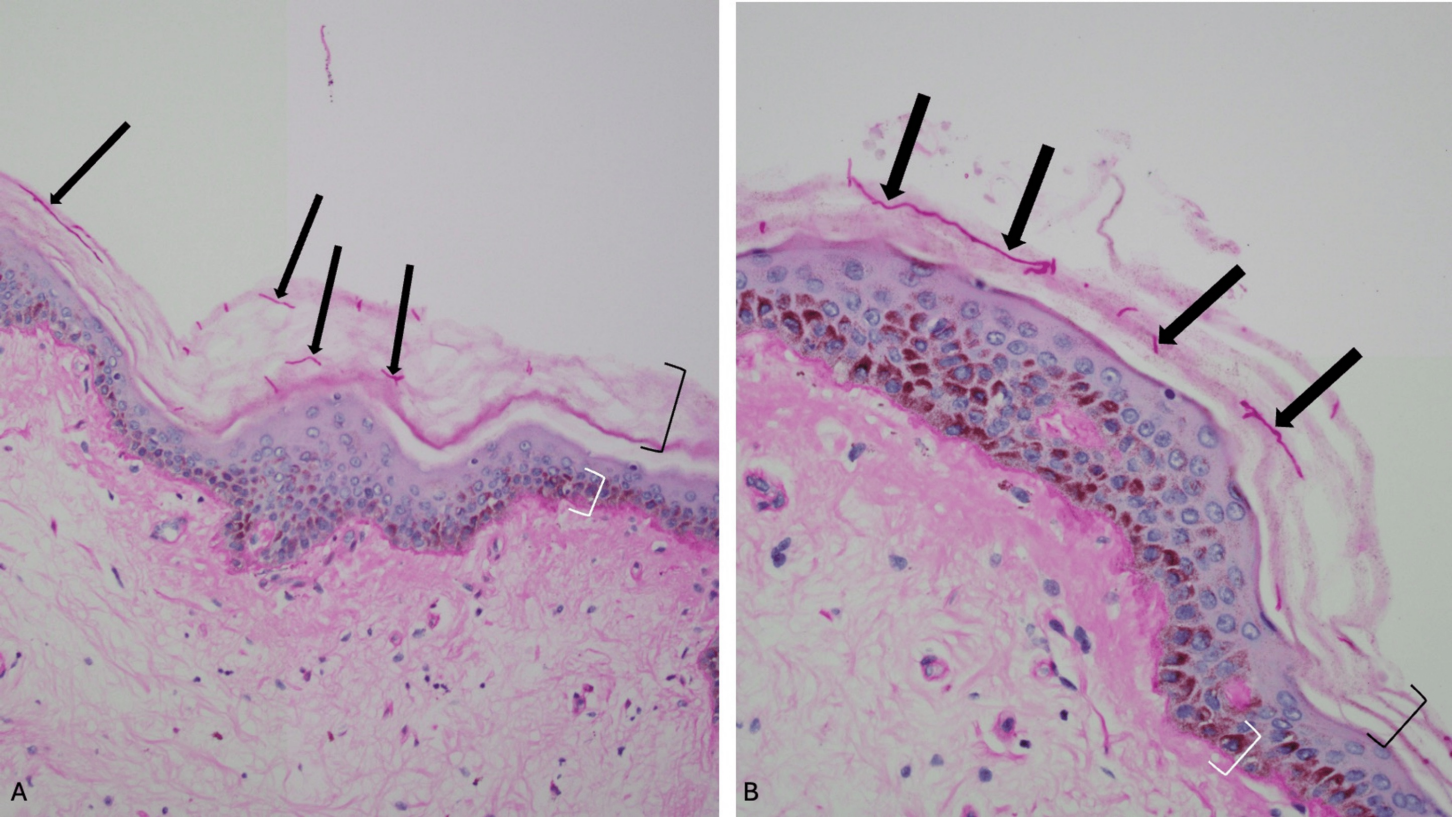
Periodic acid-Schiff-stained slides of extensive tinea corporis in Case 1. Lower (A) and higher (B) magnification views of the hyperpigmented keratotic plaque. Fungal hyphae are readily visualized after the tissue sections have been stained with periodic acid-Schiff stain (black arrows). Orthokeratosis (open black bracket) and basal layer hyperpigmentation (open white bracket) can also be observed (periodic acid-Schiff, x20 (A) and x40 (B)).

Fungal hyphae were not visualized on the hematoxylin and eosin-stained slides (Figure 3). Sections from the skin biopsy were also stained with periodic acid-Schiff stain (Figure 4). Fungal hyphae were observed in the stratum corneum, establishing the diagnosis of extensive tinea corporis.

An internal autopsy was also performed. Pertinent positive findings supported a diagnosis of hypertensive and atherosclerotic cardiovascular disease. There was cardiomegaly; the heart weighed 550 g (99th percentile). There was also hypertrophy of the left ventricular wall, which was 2.0 cm thick. In addition, there was mild to moderate calcific atherosclerosis of the aorta; the kidneys also showed moderate to severe nephrosclerosis.

Toxicology studies were performed on central blood (from the heart) and peripheral blood (from the femoral vein). The blood was positive for cocaine, benzoylecgonine (which is an inactive metabolite of cocaine), and cocaethylene (which is a toxic substance created in vivo when cocaine and ethanol are present); these findings correlate with the whitish-colored substance about the nares observed when the decedent was discovered at the location where he was found dead. The blood was also positive for delta-9-tetracannabinol (the psychoactive component of marijuana) and carboxy-tetrahydrocannabinol (the inactive, terminal metabolite of delta-9-tetracannabinol).

Toxicology studies were also performed on urine (from the bladder) and vitreous humor (from the eye). The urine was positive for cocaine, benzoylecgonine, and cocaethylene; it was also positive for carboxy-tetrahydrocannabinol. The vitreous humor was positive for cocaine and benzoylecgonine.

The cause of death and manner of death were determined based on the correlation of the autopsy findings and toxicology studies. The immediate cause of death was hypertensive atherosclerotic cardiovascular disease exacerbated by acute cocaine use, and the manner of death was accidental. The extensive tinea corporis was a coincidental finding that did not contribute to the cause of death of this individual.

Case 2

A 43-year-old Black man was found dead in a homeless encampment. His past medical history was remarkable for chronic alcoholism, chronic renal failure, and diabetes mellitus (type 2). His psychiatric history was significant for bipolar disorder and depression.

He was on numerous medications. These included amlodipine, Cogentin, diphenylhydramine, folic acid, hydroxyzine, ibuprofen, levetiracetam, loperamide, losartan, melatonin, metoprolol, olanzapine, ondansetron, Maalox, mitazapine, quetiapine, sertraline, and vitamin D. However, he was noted to be non-compliant with taking his psychiatry drugs.

A complete autopsy was performed. The cause of death was atherosclerotic heart disease. The manner of death was natural.

**Figure 5:**
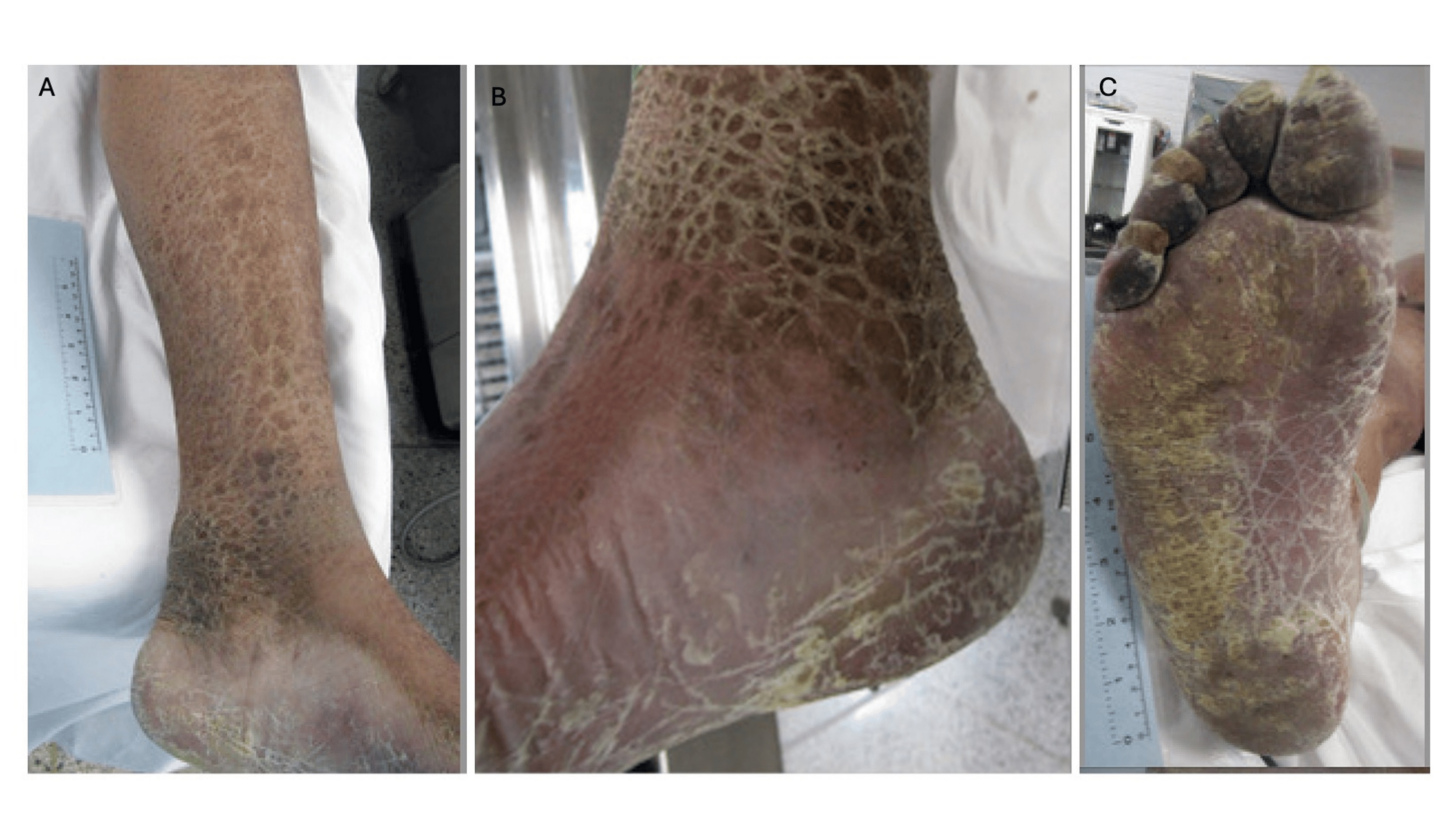
Acquired ichthyosis on the distal lower extremities and hyperkeratotic (moccasin-type) tinea pedis on the plantar foot in Case 2. The left (A) and right (B) medial distal leg of a 43-year-old Black man whose cause of death was atherosclerotic heart disease and whose manner of death was natural. The lesions on his legs were acquired and show hyperpigmented ichthyotic scaling plaques; hyperkeratotic scaling is also noted on the heel and sole of the feet. Based on the morphologic appearance of the skin lesions on his legs, the possibilities of acquired ichthyosis, dermatitis, and tinea corporis were considered in the differential diagnosis. The right plantar foot (C) shows keratotic scaly plaques and fissures; there are dystrophic changes of the toenails. The clinical diagnosis of secondary syphilis was considered in the differential diagnosis of the lesions on the soles of the feet; serologic evaluation excluded a spirochete infection.

Cutaneous examination was remarkable for ichthyotic scaling that was most prominent bilaterally on his lower legs (Figure 5). This was an acquired skin condition that had not been present when he was a younger man. He also had similar appearing areas on his shoulders, back, and thighs. In summary, scaling plaques were prominent on the man’s back, shoulders, and distal legs. The clinical differential diagnosis included acquired ichthyosis, dermatitis, and tinea corporis.

**Figure 6:**
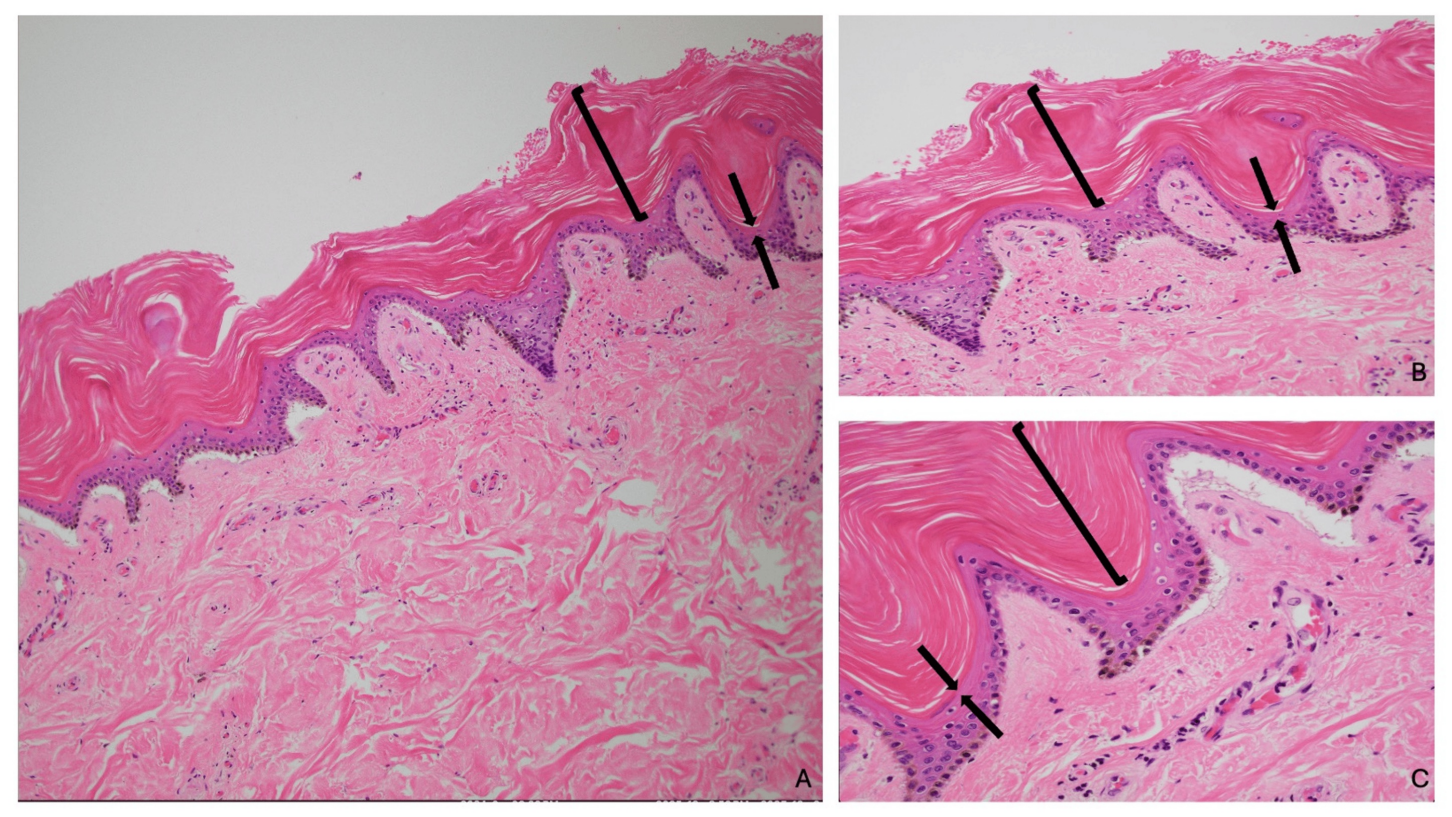
Hematoxylin and eosin-stained slides of acquired ichthyosis in Case 2. Low (A) and higher (B and C) magnification views of a biopsy from the ankle show orthokeratosis (open black bracket), demonstrating thickening of the stratum corneum with a basket weave-like appearance and no retention of keratinocyte nuclei, acanthosis (thickening of the epidermis), and absence of the granular layer, which would normally be present between the black arrows (hematoxylin and eosin stain, x10 (A); x20 (B); and x40 (C)).

The hematoxylin and eosin-stained sections from the biopsy of the right distal leg showed thickening of the stratum corneum (hyperkeratosis) consisting of a basket weave-like presentation without retention of keratinocyte cell nuclei (orthokeratosis). There was thickening of the epidermis (acanthosis), and the granular layer of the epidermis was absent. The dermis was without any inflammatory infiltrate (Figure 6). The periodic acid-Schiff stain was negative for hyphae.

Both of his feet had diffuse, dry, hyperkeratotic scaly plaques and fissures (Figure 5). This was most located on the plantar surfaces of his feet; the prominent skin lesions extended from his heels to the tips of his feet. In addition, there was dystrophy of his toenails. In summary, both feet demonstrated toenail dystrophy and thick fissured plaques on the soles. Initially, secondary syphilis was entertained as a diagnostic consideration; however, both the rapid plasma reagin (RPR) test and the fluorescent treponemal antibody absorption (FTA-ABS) test were negative. After a forensic dermatology consultation, tinea pedis (and probable concurrent onychomycosis) was added to the clinical differential diagnosis.

**Figure 7:**
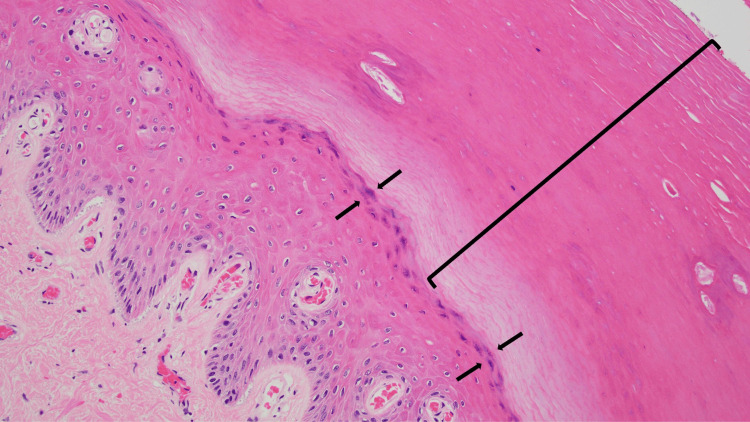
Hematoxylin and eosin-stained slides of tinea pedis in Case 2. Microscopic examination of a biopsy from the plantar foot shows hyperkeratosis, with a thickened stratum corneum consisting of compact orthokeratosis (open black bracket). In contrast to acquired ichthyosis, the granular layer is present beneath the stratum corneum (between the black arrows). The epidermis beneath the stratum corneum is also thickened (acanthosis). Fungal hyphae in the stratum corneum (open black bracket) are not readily visualized on the hematoxylin and eosin-stained sections (hematoxylin and eosin stain, x20).

**Figure 8:**
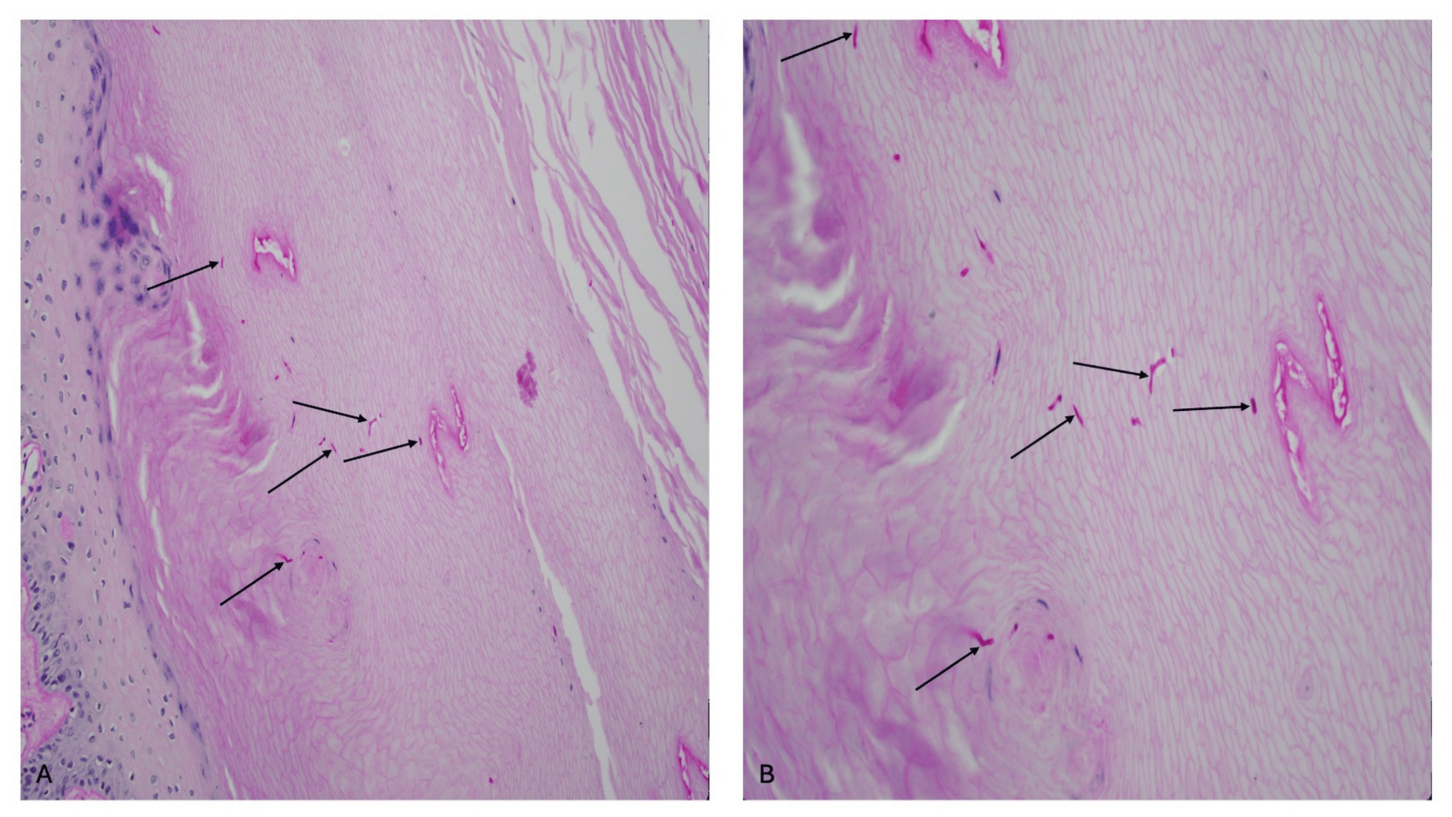
Periodic acid-Schiff-stained slides of tinea pedis in Case 2. Low (A) and higher (B) magnification views of a biopsy from the plantar foot show compact orthokeratosis overlying the epidermis. Numerous fungal hyphae (black arrows pointing to purple-staining organisms) can be observed within the hyperkeratotic stratum corneum (periodic acid-Schiff stain, x20 (A) and x40 (B)).

A biopsy of the plantar foot showed thickened stratum corneum (hyperkeratosis) and thickening of the epidermis (acanthosis). Dermatophyte hyphae could not be noticed on the hematoxylin and eosin-stained sections of the epidermis (Figure 7). However, the periodic acid-Schiff-stained sections clearly confirmed the presence of hyphae in the stratum corneum (Figure 8).

Correlation of the clinical morphology of the leg plaques, the pathological changes, and the clinical history established the diagnosis of acquired ichthyosis vulgaris. None of his medications has been previously associated with drug-induced ichthyosis. The presence of chronic renal failure was the most likely etiology for these skin changes.

Correlation of the clinical morphology of the plantar scaling and the pathological changes observed established the diagnosis of hyperkeratotic (moccasin-type) tinea pedis. The presence of nail plate changes was likely secondary to onychomycosis associated with tinea pedis. Detection of fungal hyphae on periodic acid-Schiff-stained sections confirmed the diagnosis of a dermatophyte infection of the feet.

## Discussion

Tinea infections are common. Tinea capitis, a superficial fungal infection of the scalp, is frequently encountered in children. In adults and elderly individuals, dermatophyte infections of the skin (tinea corporis) and groin (tinea cruris) may occur. Tinea pedis also develops in older men and women [1-5].

The discovery of a superficial fungal skin infection during forensic autopsies should not be unexpected. Dermatophyte infections are relatively common. Based on the incidence of dermatophyte infections of the body and feet, we suspect that they occur more frequently than is documented in autopsy reports [1,2,6,14,15].

Dermatophyte infections are among the most prevalent infectious diseases worldwide and occur in about 20% to 25% of the population [14]. Tinea corporis, in a study using data from a commercial laboratory, was diagnosed by positive culture results in 22.6% (3510 of 15,563) individuals who were clinically suspected to have the fungal infection [15]. In a study of approximately 6,800,000 Medicaid employees, 4,108 were diagnosed with tinea corporis; this resulted in an incidence rate of 79.1/10,000 person-years [14].

Tinea pedis can occur with or without accompanying onychomycosis; in a study of 169 swimming pool employees, 46% (78 of the employees) had concurrent onychomycosis and tinea pedis, and 30% had only tinea pedis [6]. Indeed, the lifetime risk of developing tinea pedis is up to 70% [1,2,6]. Also, the global prevalence of tinea pedis is estimated to be 3% [6].

In the two decedents described, the discovery of dermatophyte infections was incidental and not related to the cause or manner of death. One group of researchers specifically addressed the relationship between a forensic autopsy and fungal infection. They noted that fungi discovered postmortem occur either in the setting of infections that existed during life or as part of the decomposition process. The investigators commented that fungal organisms, such as Candida and Aspergillus, have a significant role when individuals are alive but are not often reported in decedents. The researchers also commented that fungi were more common in immunosuppressed individuals (such as patients with leukemia, sepsis, bone marrow transplantation, or heart transplantation), immunosuppressed hosts (such as patients with autoimmune diseases), and drug addicts. They concluded that in autopsy specimens, fungal infections can pose a significant challenge for the pathologist [16].

The evaluation of cutaneous findings may have important implications regarding the cause and manner of death. Gross examination of the decedent is the first aspect of a forensic autopsy. Researchers have repeatedly demonstrated that dermatologic findings at autopsy may be especially relevant for the forensic pathologist [17,18]. One group of researchers evaluated the forensic dermatopathology of skin lesions representing internal diseases discovered at autopsy. During 12 months, the evaluation of four autopsy cases had gross and microscopic skin manifestations of an internal disease that provided contributing factors to the cause of death of the individual. The conditions included brain tumor-associated signs of Leser Trelat (eruptive seborrheic keratoses), end-stage renal disease-related calciphylaxis, monoclonal gammopathy-associated with papular mucinosis, and pseudoxanthoma elasticum [17].

Another group studied the dermatology findings at autopsy. During two years, the investigators reviewed the microscopic and macroscopic dermatologic diagnoses from 389 complete autopsies. In 32% (124 cases), the external examination provided a gross autopsy diagnosis of the skin. In 10% (39 cases), a microscopic abnormality was noted. Importantly, in 4% (17 cases) of patients, the dermatologic diagnoses were identified as leading directly to the cause of death; in another 20%, the disease diagnoses of the skin contributed to the cause of death [18].

Tinea corporis may be pruritic; however, it can also be asymptomatic. It can be localized to a specific region of the body; yet, in some circumstances, it can become generalized and affect large confluent areas [1-5]. The decedent who died of a drug overdose (Case 1) had a papulosquamous dermatosis that was located on several areas of his body and presented as large confluent plaques with scaly borders.

Initially, the clinical differential diagnosis of the skin condition of the decedent (in Case 1) included dermatitis, psoriasis, mycosis fungoides, and tinea corporis. A biopsy of the lesion was performed, and the tissue sections were stained with hematoxylin and eosin, which did not provide a definitive diagnosis. The diagnostic consideration of a fungal infection was not considered until after the body of the decedent was no longer available for evaluation. Additional diagnostic methods (such as fungal culture or molecular testing) would have been advantageous to identify the specific fungal pathogen, provided the possibility of a fungal infection had initially been entertained [1-5,14-16,19].

The skin lesions (in Case 1) were subsequently evaluated by a dermatologist who suggested the possibility of tinea corporis. The microscopic sections were then stained with periodic acid-Schiff, which showed fungal hyphae that confirmed the diagnosis of tinea corporis. Therefore, the usefulness of a forensic dermatology consultation early in the evaluation of a decedent is important to consider if the dead victim has skin lesions [20].

Importantly, there are also other conditions with the same morphologic appearance of tinea corporis (Table 1) [1-5]. Some of these cutaneous conditions have subtle clinical features that might enable the investigator to establish their diagnosis based only on visual examination. However, in many circumstances, the lesions of these skin conditions so closely mimic those of tinea corporis that they may require a biopsy to determine the correct diagnosis.

**Table 1:** Disorders that can masquerade as tinea corporis. KOH prep, potassium hydroxide preparation of a skin scraping from the edge of the lesion

Disorders	Distinctive clinical features	Pathology features on hematoxylin and eosin-stained sections	References
Dermatitis (nummular eczema)	Pruritic, confluent, erythematous plaque. No central clearing. No advancing border. KOH-prep negative for fungi.	Focal parakeratosis, sometimes acanthosis or hyperkeratosis, sometimes spongiosis, and perivascular lymphohistiocytic infiltrate.	[1-5]
Erythema marginatum	Rapidly expanding (hours), asymptomatic, evanescent, pink annular patch with serpiginous raised border associated with acute rheumatic fever. KOH-prep negative for fungi.	Infiltrate of neutrophils and mononuclear cells in the superficial dermis and around blood vessels without vasculitis, and erythrocyte extravasation in later stages.	[1-5]
Erythema migrans	Rapidly expanding (days) asymptomatic patch with central clearing associated with a recent visit to a Lyme-endemic area. KOH-prep negative for fungi.	Superficial and perivascular lymphocytic infiltrate, plasma cells at the periphery of the lesion, and eosinophils at the center of the lesion.	[1-5]
Fixed drug eruption	Recurrent, at the same location, erythematous round or oval plaque; bullae can be present or absent. KOH-prep negative for fungi.	Dense, dermal band-like infiltrate of lymphocytes (often with neutrophils and eosinophils) along the basal layer of the epidermis, vacuolar (hydropic) degeneration of the epidermal basal cells, necrotic keratinocytes throughout the epidermis, occasionally subepidermal blistering, and incontinence of pigment and melanophages in the upper dermis.	[1-5]
Granuloma annulare	Annular arrangement of asymptomatic dermal papules. KOH-prep negative for fungi.	Epidermis normal, palisading of histiocytes and giant cells around small foci of mild collagen degeneration and mucin accumulation, and often single filing of inflammatory cells between collagen bundles.	[1-5]
Lupus erythematosus (cutaneous discoid)	Hypopigmented and hyperpigmented plaque with telangiectasias often on sun-exposed sites. KOH-prep negative for fungi.	Hyperkeratosis, follicular plugging, epidermal atrophy or hyperplasia, sometimes colloid bodies in the lower epidermis or papillary dermis, liquefaction degeneration of the basal layer, melanin incontinence, thickened basement membrane, upper dermal edema, increased mucin in the dermis, and perivascular and periadnexal (sometimes lichenoid) mononuclear infiltrate.	[1-5]
Pityriasis rosea	Single plaque (herald patch) followed by eruption of plaques (with collarette of scale on periphery) distributed in a fir tree pattern on the back. KOH-prep negative for fungi.	Focal parakeratosis, sometimes mild acanthosis, spongiosis, perivascular lymphohistiocytic infiltrate, and sometimes focal extravasated red blood cells.	[1-5]
Psoriasis vulgaris	Annular erythematous, well-demarcated plaques on the body; scalp involvement and nail dystrophy may also be present. KOH-prep negative for fungi.	Confluent parakeratosis, neutrophils in the stratum corneum (Munro microabscesses), hypogranulosis, suprapapillary thinning of the epidermis, regular acanthosis, dilated capillaries in dermal papillae, and perivascular lymphohistocytic infiltrate.	[1-5]
Tinea corporis	Annular plaque with expanding raised scaling border and central clearing. KOH-prep positive for fungal hyphae.	Often, neutrophils are found in the stratum corneum, sometimes parakeratosis, sometimes spongiosis or intraepidermal vesicles, sometimes folliculitis, variable inflammatory response (skin may appear normal), or diffuse mixed infiltrate of lymphocytes, histiocytes, neutrophils, or eosinophils, and fungal hyphae present in the stratum corneum (or in follicles), difficult to visualize.	[1-5]

Microscopic examination of the hematoxylin and eosin-stained sections of tinea corporis may be misinterpreted as normal skin. Periodic acid-Schiff staining is negative in all the disorders that mimic tinea corporis, except for discoid cutaneous lupus erythematosus, in which the periodic acid-Schiff stain will highlight the thickened basement membrane between the epidermis and dermis. Yet, staining with periodic acid-Schiff stain will only demonstrate fungal hyphae in the stratum corneum in living or dead individuals with a dermatophyte fungal skin infection, such as tinea corporis [1-6,14,15].

The man who died from atherosclerotic heart disease had several other medical problems (Case 2); he presented with diffuse hyperkeratotic scaling plaques on the soles of his feet. The clinical presentation of his soles raised concern regarding the possibility of secondary syphilis plantar cutaneous lesions. However, his serologic evaluation for syphilis was negative.

In addition, a biopsy of a skin lesion from the sole of his foot did not show any plasma cells in the dermis. Also, the periodic acid-Schiff stain of the plantar biopsy demonstrated hyphae, confirming the diagnosis of tinea pedis. Several conditions can be considered in the clinical differential diagnosis of tinea pedis, including erythrasma, juvenile plantar dermatosis, psoriasis, and rarely secondary syphilis [4].

The man from Case 2 also had well-defined small plaques on his extremities. These were acquired ichthyotic lesions, presumptively related to his chronic renal disease. A hereditary condition exists with skin lesions that have an identical clinical morphology and pathology changes to those of acquired ichthyosis [11-13]. In addition to chronic renal disease, there are several conditions and medications that can be associated with acquired ichthyosis (Table 2) [7-11].

**Table 2:** Conditions associated with acquired ichthyosis vulgaris.

Condition	References
Autoimmune/inflammatory diseases: dermatomyositis, eosinophilic fasciitis, graft-versus-host disease, sarcoidosis, systemic sclerosis, and systemic lupus erythematosus overlap, and systemic lupus erythematosus	[8-12]
Drugs: allopurinol, benzophene, bevacizumab, brodalumab, butyrophenone, cholesterol lowering agents (azacosterol hydrochloride, niacin, nicotinic acid, triparanol and 3-hydroxy-3-methylglutaryl coenzyme A reductase inhibitors such as pitavastin and pravastatin), cimetidine, clofazimine, dixyrazine, hydroxyurea, isoniazid, losartan, maprotiline, nafoxidine, ponatinib, and retinoids (acitretin)	[8-12]
Infections: Acquired immunodeficiency syndrome, human immunodeficiency virus, human T-lymphotrophic virus-I, human T-lymphotrophic virus-I associated myelopathy/tropical spastic paraparesis, human T-lymphotrophic virus-II, and mycobacteria (Mycobacteria lepra and Mycobacteria tuberculosis)	[8-12]
Malignant hematologic malignancies: leukemias (adult T cell leukemia/lymphoma and chronic myelogenous), lymphomas (anaplastic large cell, cutaneous T-cell, granulomatous slack skin, Hodgkin’s, non-Hodgkin’s, intravascular diffuse large B cell, and mycosis fungoides), multiple myeloma, myelodysplastic syndrome, and polycythemia vera	[7-12]
Malignant solid tumors-carcinomas: breast, cervix, kidney (renal cell, or transitional cell), lung, ovary, parotid gland (undifferentiated), and stomach (adenocarcinoma)	[8-12]
Malignant solid tumors-sarcomas: hemangiopericytoma (osseous), Kaposi’s sarcoma, leiomyosarcoma (intestinal), lymphosarcoma, rhabdomyosarcoma, reticulum cell sarcoma, and spindle cell sarcoma	[8-12]
Metabolic diseases: chronic hepatic dysfunction, chronic renal failure, diabetes mellitus (type I), hypothyroidism, hyperthyroidism, hypopituitarism, and thyroiditis (autoimmune)	[8-12]
Neurologic intervention: sympathectomy	[8-12]
Nutritional diseases: essential fatty acid deficiency, malabsorption (celiac disease, kwashiorkor, and pancreatic insufficiency), malnutrition, pellagra, vitamin A deficiency, vitamin deficiency, and zinc deficiency	[8-12]
Other causes: chronic kava plant ingestion, lymphomatoid papulosis, porphyria cutanea tarda, post-bone marrow transplant, and radiotherapy	[8-12]

Hematoxylin and eosin staining of the papulosquamous lesion of the man’s ankles (Case 2) demonstrated hyperkeratosis (consisting of an increased thickness of the stratum corneum composed of orthokeratosis) and acanthosis (demonstrated by an increased thickness of the epidermis beneath the stratum corneum). Importantly, there was a distinct absence of the granular layer of the epidermis; normally, this layer of the epidermis is present immediately below the stratum corneum. Since the man’s condition was not hereditary, these findings established the diagnosis of acquired ichthyosis vulgaris [7-13].

In addition to dermatophyte infections and ichthyosis vulgaris, there are numerous cutaneous conditions that can present with either localized or diffuse plaques [1-13]. It is crucial to emphasize that the coexistence of two distant conditions with overlapping morphologies may lead to misinterpretation; failure to differentiate the conditions may result in a diagnostic pitfall in forensic pathology. In these circumstances, visual inspection may not be adequate to establish the diagnosis with certainty; also, in some individuals (such as Case 2), more than one skin condition with a papulosquamous morphology can be present. In these situations, the forensic pathologist needs to perform a biopsy of the lesion not only for routine hematoxylin and eosin staining but also for staining the biopsy specimen with a stain that can more readily demonstrate fungal organisms (such as the periodic acid-Schiff stain) to confirm or exclude the possibility of a superficial dermatophyte fungal skin infection.

This paper has certain inherent limitations, since it is a case report. Specifically, the observations have limited generalizability. There is also an absence of longitudinal clinical correlation.

## Conclusions

A forensic autopsy may include not only an external examination but also an internal evaluation. A comprehensive inspection of the skin, mucous membranes, hair, and nails may reveal extensive findings. The presence of dermatology-related lesions can be related to primary skin disorders, neoplasms, or infections; indeed, the lesions may or may not be associated with the cause of death of a decedent. In fact, it is not uncommon for unusual skin findings at forensic autopsy to represent incidental findings, as illustrated by the cases presented. Superficial fungal infections can present asymptomatically on the skin, hair, and/or nails; the dermatophyte-related lesions can result in extensive involvement of the body and masquerade as other cutaneous conditions. Acquired conditions of the skin, such as ichthyosis, can also be diffuse and present with prominent involvement of the body surface. Two male decedents presented with dermatophyte infections of their skin whose morphology mimicked other cutaneous conditions; the cutaneous features of the superficial fungal skin infection of the men were selected for presentation in this paper because of their illustrative value. One man had extensive tinea corporis that was initially misinterpreted as possibly being either another papulosquamous condition (such as psoriasis or dermatitis) or a cutaneous neoplasm such as cutaneous T-cell lymphoma. The other man had severe hyperkeratotic (moccasin-type) tinea pedis that initially prompted an evaluation to exclude plantar lesions of secondary syphilis; in addition, he also had a second skin condition, acquired ichthyosis, that presented as cutaneous plaques. The diagnosis of superficial fungal skin infections was established after microscopic examination of the skin biopsies of the lesions; importantly, the fungal hyphae could not be readily visualized on the hematoxylin and eosin-stained sections, but were easily identified when the tissue was stained with periodic acid-Schiff stain, which demonstrates fungal organisms. Also, it is essential to consider that an individual may have more than one skin condition concurrently present, such as the man with acquired ichthyosis (demonstrating an absence of both the granular layer and fungal hyphae) and tinea pedis (which had the presence of both the granular layer and fungal organisms in the stratum corneum). In summary, the forensic pathologist should consider performing a skin biopsy (and staining the tissue not only with hematoxylin and eosin but also periodic acid-Schiff stain) for the evaluation of any papulosquamous skin conditions in a decedent. Consultation with a dermatologist can be especially valuable when developing a differential diagnosis for such lesions.

## References

[1] Odom R (1993). Pathophysiology of dermatophyte infections. J Am Acad Dermatol.

[2] Rupke SJ (2000). Fungal skin disorders. Prim Care.

[3] Gupta AK, Chaudhry M, Elewski B (2003). Tinea corporis, tinea cruris, tinea nigra, and piedra. Dermatol Clin.

[4] Kelly BP (2012). Superficial fungal infections. Pediatr Rev.

[5] Diep D, Calame A, Cohen PR (2020). Tinea corporis masquerading as a diffuse gyrate erythema: case report and a review of annular lesions mimicking a dermatophyte skin infection. Cureus.

[6] Leung AK, Barankin B, Lam JM, Leong KF, Hon KL (2023). Tinea pedis: an updated review. Drugs Context.

[7] Dalcin D, Beecker J (2018). Acquired Ichthyosis. J Cutan Med Surg.

[8] Haber R, Feghali J, Nadir U, Yi MD, Cahn BA (2023). Acquired ichthyosis: a clinical review. Arch Dermatol Res.

[9] Patel N, Spencer LA, English JC 3rd, Zirwas MJ (2006). Acquired ichthyosis. J Am Acad Dermatol.

[10] Cather JC, Cohen PR (1999). Ichthyosiform sarcoidosis. J Am Acad Dermatol.

[11] Okulicz JF, Schwartz RA (2003). Hereditary and acquired ichthyosis vulgaris. Int J Dermatol.

[12] DiGiovanna JJ, Robinson-Bostom L (2003). Ichthyosis: etiology, diagnosis, and management. Am J Clin Dermatol.

[13] Jaffar H, Shakir Z, Kumar G, Ali IF (2023). Ichthyosis vulgaris: an updated review. Skin Health Dis.

[14] Sajewski ET, Benedict K, Caplan AS, Lipner SR, Gold JA (2026). Tinea corporis and tinea cruris incidence, risk factors, and treatments in a cohort of 6.8 million patients with Medicaid, United States. Med Mycol.

[15] Zarzeka D, Benedict K, McCloskey M, Lockhart SR, Lipner SR, Gold JA (2024). Current epidemiology of tinea corporis and tinea cruris causative species: analysis of data from a major commercial laboratory, United States. J Am Acad Dermatol.

[16] Roll P, Reichenpfader B, Grabuschnigg P (2007). Forensic autopsy and fungi. Wien Med Wochenschr.

[17] Kovarik CL, Stewart D, Cockerell CJ, Barnard JJ (2005). Forensic dermatopathology and internal disease. J Forensic Sci.

[18] Cocks M, Sander I, Crain B (2018). Frequency of dermatologic findings at autopsy. J Forensic Sci.

[19] Wickes BL, Wiederhold NP (2018). Molecular diagnostics in medical mycology. Nat Commun.

[20] Cohen PR (2025). Forensic dermatology is an integral subspecialty of forensic medicine. Cureus.

